# Self-Adaptive Telemedicine Specialist Recommendation Considering Specialist Activity and Patient Feedback

**DOI:** 10.3390/ijerph19095594

**Published:** 2022-05-05

**Authors:** Wei Lu, Yunkai Zhai

**Affiliations:** 1 School of Management Engineering, Zhengzhou University, Zhengzhou 450001, China; luwei622@163.com; 2 National Engineering Laboratory for Internet Medical Systems and Applications, Zhengzhou 450052, China

**Keywords:** specialist recommendation, telemedicine, activity, feedback adjustment, cold start

## Abstract

**Purpose:** With the rapid development of medical informatization, information overload and asymmetry have become major obstacles that limit patients’ ability to find appropriate telemedicine specialists. Although doctor recommendation methods have been proposed, they fail to address data sparsity and cold-start issues, and electronic medical records (EMRs), patient preferences, potential interest of service providers and the changes over time are largely under-explored. Therefore, this study develops a self-adaptive telemedicine specialist recommendation method that incorporates specialist activity and patient utility feedback from the perspective of privacy protection to fill the research gaps. **Methods:** First, text vectorization, view similarity and probabilistic topic model are used to construct the patient and specialist feature models based on patients’ EMRs and specialists’ long- and short-term knowledge backgrounds, respectively. Second, the recommended specialist candidate set and recommendation index are obtained based on the similarity between patient features. Then, the specialist long-term knowledge feature model is used to update the newly registered specialist recommendation index and the recommended specialist candidate set to overcome the data sparsity and cold-start issues, and the specialist short-term knowledge feature model is adopted to extend the recommended specialist candidate set at the semantic level. Finally, we introduce the specialists’ activity and patients’ perceived utility feedback mechanism to construct a closed-loop adjusted and optimized specialist recommendation method. **Results:** An empirical study was conducted integrating EMRs of telemedicine patients from the National Telemedicine Center of China and specialists’ profiles and ratings from an online healthcare platform. The proposed method successfully recommended relevant and active telemedicine specialists to the target patient, and increased the recommended opportunities for newly registered specialists to some extent. **Conclusions:** The proposed method emphasizes the adaptability and acceptability of the recommended results while ensuring their accuracy and relevance. Specialists’ activity and patients’ perceived utility jointly contribute to the acceptability of recommended results, and the recommendation strategy achieves the organic fusion of the two. Several comparative experiments demonstrate the effectiveness and operability of the hybrid recommendation strategy under the premise of data sparsity and privacy protection, enabling effective matching of patients’ demand and service providers’ capabilities, and providing beneficial insights for data-driven telemedicine services.

## 1. Introduction

Telemedicine, an essential means of online assisted diagnosis and treatment relying on medical institutions, has flourished because of its ability to solve many problems faced by offline healthcare, such as time wastage, geographical inconvenience, and uneven service capacity [1]. Especially during the outbreak of the COVID-19 pandemic in China, in order to effectively comply with the principle of “local management”, most suspected and confirmed cases were treated by expert teams through telemedicine in their respective locations, which reduced the risk of cross-infection of patients and played a more prominent role [2]. Since the start of the COVID-19 pandemic, the telemedicine industry has experienced significant growth resulting in a 154% increase in appointments compared to the same period in 2019 [3]. The industry is expected to be worth $266.8 billion by 2026 [4,5]. Telemedicine has collected and accumulated a large amount of clinical data representing patients’ health conditions during its use, significantly increasing the digital information available for patient-oriented decision making, and making it possible for data-driven personalized healthcare services.

The rapid development of Internet capabilities has led to public changes in attitudes toward information seeking and has affected the way patients seek doctors [6]. Increasing needs for health information and changes in information-seeking behavior can be observed globally [7]. A study conducted in 2012 by Pew Research Center reported that 59% of adults in the United States search for health information online and tend to be active participants in the decision-making process and engage in discussions about relevant issues [8]. The main concern of patients is to find the most professional medical specialists to solve their healthcare issues [9]. However, due to the rapid growth of information and the lack of professional medical backgrounds, patients are confused when they are exposed to similar, excessive, vague and misleading information. Information overload and knowledge trek caused by irrelevant information are major obstacles for patients to take appropriate actions. It is difficult for patients to find the appropriate specialist to solve their health problems in a timely and effective manner, as well as facing huge time and search costs, even resulting in wasted medical resources and reduced treatment efficiency [10]. It can be challenging for patients to select the right specialist for themselves, especially without a suitable matching mechanism [11]. Currently, most existing practices use the manual recommendation by the requesting physician or scheduler to select a consultation specialist for the patient. However, as the consultations increase, manual recommendations cannot guarantee the professionalism and quality of medical services, and coupled with the asymmetry of medical information, it tends to cause the distrust of patients, resulting in negative effects on the doctor–patient relationship and patient satisfaction. Meanwhile, the barriers between a rapidly changing institutional environment and increasing patient autonomy complicate the doctor–patient matching. It seems necessary to explore an intelligent telemedicine specialist recommendation method to recommend the appropriate specialists for patients.

Personalized recommendation techniques can help users effectively deal with the information overload and knowledge trek [12] by recommending items that satisfy their interests and needs, and have been widely employed in Amazon’s book promotions, Netflix’s movie suggestions, Pandora’s music recommendations, and many other fields [13] to facilitate users’ product selection process [14]. Related techniques and methods have been applied to the recommendation of doctors and other medical resources, and have become a driving force in the delivery of patient-centered personalized healthcare services [15], helping patients and telemedicine schedulers filter out a large number of irrelevant specialists, and quickly and accurately find telemedicine specialists that meet patients’ needs at a professional level. This can benefit patients and telemedicine providers by reducing patient search costs, assisting in medical decision making, ensuring the effective value in healthcare delivery.

At present, recommendation methods for medical resources are mainly divided into two categories: one is the doctor recommendation based on a voting scoring mechanism, which essentially assigns a static or dynamic comprehensive authoritative ranking to doctors and recommends doctors for patients according to the ranking. For example, Guo et al. [16] identified key opinion leaders by integrating their ranking features with professional footprints, and formed recommended doctor rankings. Most online healthcare websites also use the above strategy to develop doctor rankings and recommend the same doctors for all patients [17]. However, this strategy ignores text information that records doctor–patient information and reflects a large amount of semantic information, for example the description of patients’ health conditions in electronic medical records (EMRs). It fails to personalize the matching of patients’ needs and doctors’ capabilities, and affects the service quality and efficiency, thereby reducing patient satisfaction. The second is similarity-based medical resource recommendation, which uses the ideas of collaborative filtering, content-based recommendation and deep learning to recommend relevant medical resources for patients based on the similarity between patients’ questions and doctors’ labels, such as Zaman et al. [18] who used semantics-enhanced social networking technology to make recommendations for users with similar health problems to accelerate their recovery and improve healthcare outcomes. Lee et al. [19] proposed a healthcare service recommendation framework considering each user’s health profile and contextual information to arrange healthcare services in accordance with the medical similarity between the user and the service. Chen et al. [20] clustered disease symptoms and introduced the Apriori algorithm for correlation analysis of disease diagnosis and treatment rules to recommend appropriate medical options for patients and inexperienced physicians. However, most existing studies have been conducted from a social network perspective and require the submission of significant additional user behavior data, with less consideration of data sparsity. Few patients vote or rate different types of healthcare items in telemedicine contexts. Meanwhile, such information is often difficult to obtain during practical consultations due to privacy protection, and the data sparsity issue leads to difficulties in capturing patients’ preferences for doctors and poor recommendation performance [21]. In addition to the mentioned problems, existing research has performed recommendations only in terms of patients’ preferences for doctors’ features and the matching ability between doctor–patient features, ignoring patients’ preferences for different recommendation ways [17] and changes in doctors’ activity over time, leading to inappropriate recommendations and increased matching costs, thereby affecting the overall system performance. Additionally, the cold-start problem has also been insufficiently considered. Newly registered physicians have a limited chance of being recommended due to a lack of historical data.

To solve the above issues, this study proposes a novel approach for telemedicine specialist recommendation and answers the following four questions:

- How to effectively model patient and specialist features with privacy-preserving policies and address data sparsity and cold-start issues?
- How to measure specialists’ activity and its change over time, and incorporate them into the recommendation index?
- How to construct a self-adaptive specialist recommendation model that considers patients’ preferences for different recommendation ways?
- How to verify the effectiveness and rationality of the recommendation strategy?

In response, the specialist recommendation in this study operates under the premise of privacy protection, where patients are considered as independent individuals. We characterize user features with patients’ EMRs and specialists’ long- and short-term knowledge background, and propose an automated specialist recommendation with feedback adjustment that incorporates specialists’ activity. Specifically, topic modeling and feature modeling are used to model patient and specialist features. The specialist recommendation index and recommended specialist candidate set are generated based on the similarity between patient features. Then, the similarity between knowledge views for specialist long-term knowledge features is calculated to update the newly registered specialist recommendation index and recommended specialist set, and the semantic correlation between specialist short-term knowledge features is obtained based on the topic space to extend the recommended specialist candidate set at the semantic level. This achieves feature modeling of patients and specialists and solves the data sparsity and cold-start issues, answering the first research question of this paper. Second, the activity index of specialists is proposed and its time window is considered to develop a specialist recommendation index that integrates the activity, which solves the second question of this research. Patient utility feedback is then incorporated into the recommendation framework, and a self-adaptive telemedicine specialist recommendation approach with feedback adjustment is developed combining with the attention mechanism, addressing the third research question. The fourth research question is answered by an empirical study and several comparative experiments.

The rest of this paper is organized as follows. Section 2 describes the framework of the recommendation approach proposed in this paper, which consists of four modules. The proposed telemedicine specialist recommendation approach and its construction process are introduced in Section 3. Section 4 performs an empirical study to evaluate the proposed approach and discusses the evaluation results. Section 5 summarizes this study by outlining its major contributions and possible future works.

## 2. Telemedicine Specialist Recommendation Framework

**Figure 1:**
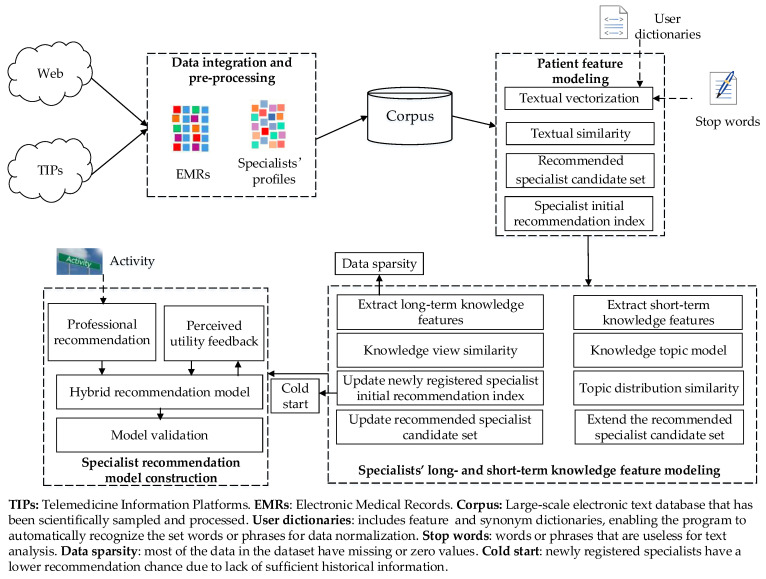
Telemedicine specialist recommendation framework.

To address the above issues, this paper constructs a telemedicine specialist recommendation framework composed of four modules, as shown in Figure 1.

- Data integration and pre-processing: multi-source heterogeneous data are collected, extracted and integrated, which performed operations such as de-duplication, consistency and integrity checks, and then fill rules are constructed to enhance data integrity and reliability.
- Patient feature modeling: the stop words list, and feature and synonym dictionaries are introduced to perform text segmentation on the reliable corpus for text vectorization representation of patients’ EMRs. Then the similarity between patients’ EMRs is calculated to find experienced specialists to be included in the recommended candidate set, and the initial recommendation index of specialists is obtained accordingly.
- Specialists’ long- and short-term knowledge feature modeling: constructing a specialists’ long-term knowledge feature model based on their long-term accumulated knowledge profiles, and extending the recommended specialist set and updating the initial recommendation index according to the similarity of specialists’ long-term knowledge views. Then, the short-term knowledge feature topic model is constructed based on EMRs diagnosed by the specialist. The short-term knowledge features of specialists are mapped to the latent topic space, and the recommended specialist candidate set is extended by calculating the semantic correlation between specialist features in the same topic space.
- Specialist recommendation model construction: we propose a specialist recommendation index that incorporates specialists’ activity to bias the recommendation results toward specialists with higher motivation, and further integrate patient-perceived utility feedback into the recommendation method to realize a self-adaptive specialist recommendation model with feedback adjustment. The effectiveness of the model is eventually verified by comparative experiments.

## 3. Telemedicine Specialist Recommendation Approach

**Figure 2:**
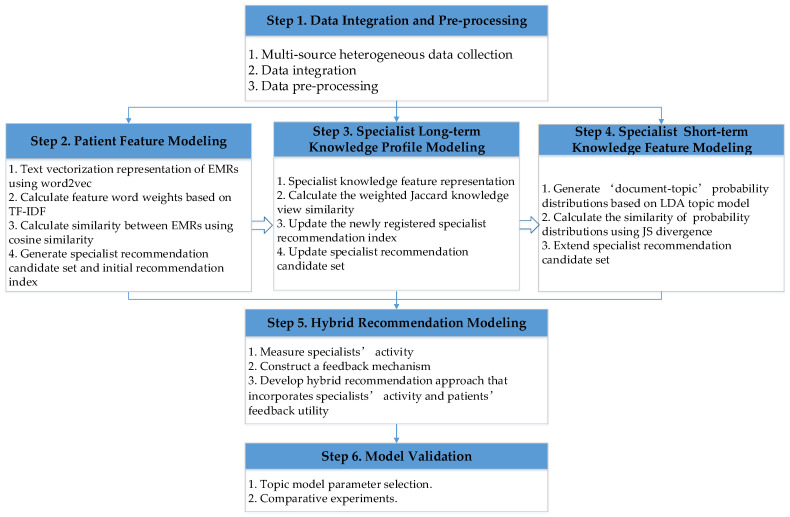
Self-adaptive telemedicine specialist recommendation approach flowchart.

In this paper, a self-adaptive telemedicine specialist recommendation approach with feedback adjustment that incorporates specialists’ activity and patients’ feedback utility is constructed, and its flowchart is shown in Figure 2.

### 3.1. Data Integration and Pre-Processing

Since the data comes from dual channels, namely the business data accumulated in the database of the telemedicine information platform and the specialists’ profiles published on the online healthcare platform, the raw data are relatively crude. So pre-processing operations need to be performed before text mining. The specific steps are as follows.

**Step 1.** Extracting, integrating and storing relevant data, and performing operations such as standardization, de-duplication and missing value-filling to ensure the integrity and consistency of the data, thereby forming a reliable corpus.

**Step 2.** Medical vocabulary is highly specialized and expressed in different ways. It is necessary to uniformly replace synonymous disease names and non-standard writing in the medical field.

**Step 3.** User dictionaries are established to correctly identify specialized vocabulary in the medical field, and the stop words list is used to filter out meaningless words, numbers, and symbols.

### 3.2. Patient Feature Modeling

EMRs describe the features of patients’ disease in specialized terms. For the highly professional medical terms, this paper introduces the synonym dictionary and feature word dictionary for text normalization and feature word extraction. This strategy enhances the representational ability of the text, enables professional terms to be correctly identified and classified, thereby compressing the dimension of feature space and improving the efficiency of data processing. Contextual information is used to convert high-dimensional sentences into low-dimensional real vectors to realize the text segmentation and vectorized representation, and to form the patient feature model by extracting keywords. The specific steps are as follows.

**Step 1.** Text vectorization representation. Based on the introduction of feature and synonym dictionaries and the stop words list, the text is converted into a feature word set consisting of words using the text segmentation technique. Then the vector of each keyword is calculated using the word2vec model, and the average of the non-repeating word vectors is taken to aggregate the sentence vector to represent the final vector of the text. For example, the EMR of patient $p_{i}$ consists of f feature words, its normalized representation is $p.feature\_profile=\{w_{k}|w_{k}\in d_{i},k=1,2,\ldots ,f\}$. $d_{i}$ denotes the EMR of $p_{i}$, $w_{k}$ denotes the k-th feature word, whose corresponding word vector is $v_{k}=\{v_{k1},v_{k2},\ldots ,v_{kp}\}$.

**Step 2.** Feature word weight calculation. Term Frequency (TF) refers to how often a given word appears in the text, while Inverse Document Frequency (IDF) measures the importance of the word [22]. The larger the value of TF-IDF, the more important the feature word is, and vice versa. Hence, this paper adopts the TF-IDF value as the weight to describe the importance of feature words, and its specific expression is shown in Equations (A1)–(A3) in Appendix A. Therefore, the feature vector of $d_{i}$ is represented as:

(1) $$d_{i}=\frac{1}{f}\sum_{k=1}^{f}v_{k}\times tf-idf(w_{k},d_{i})$$

**Step 3.** Text similarity measure. The similarity $Sim(d_{i},d_{j})$ between vector texts can be calculated using cosine similarity. Cosine similarity is one of the easiest and most effective methods of calculating vector similarity, and its equation is shown as:

(2) $$Sim(d_{i},d_{j})=\cos\theta =\frac{d_{i}\times d_{j}}{\sqrt{{\left(d_{i}\right)}^{2}}\times \sqrt{{\left(d_{j}\right)}^{2}}}$$

**Step 4.** Recommended specialist candidate set generation. The initially recommended specialist candidate set is composed of specialists who treat similar patients, and the highest similarity value is considered as the specialist’s initial recommendation index ini_score. To recommend high-quality specialists, it is necessary to return specialists with high similarity, but setting the similarity threshold will limit the recommended results since patients suffer from uncommon diseases in telemedicine. Therefore, the following two conditions are set up in this paper: (a) Counting all similar specialists and using the highest similarity of their similar patients as the initial recommendation index and sorting them in descending order; (b) Top-10 are selected from the ranking results and included in the candidate set.

### 3.3. Specialist Long- and Short-Term Knowledge Feature Modeling

Short-term knowledge features reflect specialists’ recent concerns and interests, while long-term knowledge features express their persistent traits and are relatively stable [23]. The combination of the above two can describe the knowledge background of specialists comprehensively and improve the accuracy and scientificity of recommendations. The database of EMRs that the specialist has diagnosed reflects his/her interest domain over a certain period of time, while the specialist’s profile represents his/her long-term accumulated experience.

The recommendation strategy that determines the recommended specialist candidate set based on the similarity of patients’ EMRs can only find specialists who have diagnosed conditions similar to those of the target patient, but cannot select specialists who specialize in treating similar diseases. Moreover, specialists who are newly registered in the system or have a small number of diagnosed cases (collectively called “newly registered specialists”) are less likely to be recommended due to insufficient business volume and historical data support.

To solve the cold-start problem of newly registered specialists, this paper conducts knowledge view similarity measurement for the long-term knowledge profiles of specialists, and gives the newly registered specialists a newly recommended index to increase their recommended opportunities. Additionally, the LDA topic probability model can effectively identify doctors who specialize in treating similar diseases from the semantic level, which greatly reduces the scale and time cost of finding similar doctors.

#### 3.3.1. Knowledge View-Based Long-Term Knowledge Feature Modeling

##### Feature Representation

In response to the diversity of knowledge, it is given different attributes according to its domain and research expertise. For example, there is a many-to-many relationship between doctors and diseases, namely, a doctor might be specialized in multiple diseases, and the disease can also be specialized by multiple doctors. The doctor’s expertise can be represented by a vector and takes the value of {0,1}; 1 means that the doctor is specialized in the disease, otherwise, not specialized in the disease. Therefore, the similarity between specialists’ long-term knowledge profiles is characterized by the view similarity of specialists’ knowledge attributes, for which the specialist knowledge attribute matrix is constructed as shown in Table 1.

The incompleteness of specialist profile information makes the feature model face the problem of data sparsity. A lower similarity would result if attributes that all specialists have simultaneously are used as fill values, that is, if all other specialists take the value of 1 under a certain attribute, the missing value is filled as 1. To maintain fairness, this paper adopts the frequency statistics for the missing value-filling. Specifically, let $a_{jp}$ be a missing value, namely the p-th knowledge attribute of the specialist $S_{j}$ is missing, and if $\sum_{j=1}^{n}a_{jp}/\left|S\right|\geq 0.5$, then $a_{jp}=1$, otherwise $a_{jp}=0$, where |S| is the total number of specialists, and the specialists’ long-term knowledge features are described as $d.feature\_profile=\{a_{jp},j=1,2,\ldots ,n;p=1,2,\ldots ,g\}$. 

##### Recommended Index Update

The knowledge structure of specialists is constructed according to their expertise in diseases, and their matching ability is predicted by calculating the similarity of knowledge views. Based on the attribute characteristics of knowledge, we use Jaccard similarity coefficient to calculate the attribute similarity between knowledge. Meanwhile, based on the difference in contribution and importance of different knowledge, different knowledge is distinguished by weights to obtain the weighted Jaccard knowledge view similarity coefficient $Sim_{Knowledge}(j,h)$, which indicates the long-term knowledge similarity between specialist $S_{j}$ and $S_{h}$, and its calculation process is shown in Appendix B.

Moreover, we set the specialist similarity threshold to 0.7 to return specialists with high similar knowledge profiles, that is, if $Sim_{Knowledge}\geq 0.7$, then their index is returned. After that, the recommendation index of newly registered specialists is updated to $ini\_score_{j}^{\prime }=ini\_score_{j}+\frac{1}{q}\sum_{h=1}^{q}ini\_score_{h}\times Sim_{Knowledge}(j,h)$, where $ini\_score_{h}$ denotes the initial recommendation index of similar specialists who meet the threshold requirement, and q denotes the number of similar specialists for newly registered specialists. Finally, the recommended specialist candidate set is updated according to the new recommendation index.

#### 3.3.2. LDA-Based Short-Term Knowledge Feature Modeling

##### LDA Topic Model

LDA, an unsupervised generative probabilistic approach for corpus modeling [24], is the most common approach for topic modeling [25]. The basic idea [26] is that documents are represented as random mixtures over latent topics, where the topics consist of feature words with a specific probability distribution. LDA divides the high-dimensional document-word matrix into two: low-dimensional document-topic matrix and topic-word matrix according to the probability distribution of documents and words to obtain the topic distribution of documents. The generation process of a text can be formally expressed as follows.

**Figure 3:**
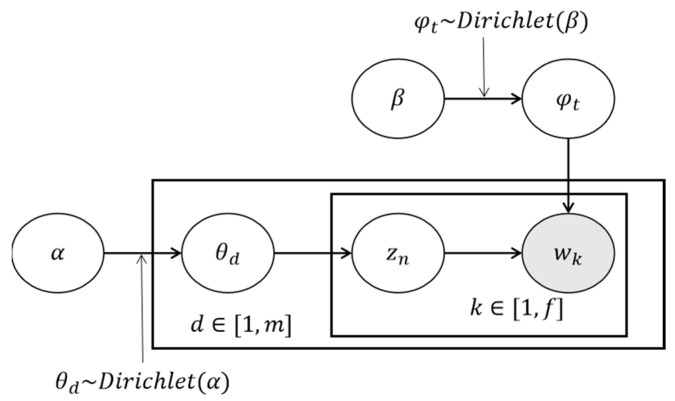
LDA probabilistic model.

(1) Choose a multinomial distribution $\theta_{d}$ for the document d from a Dirichlet distribution with parameter α, i.e., $\theta_{d}∼Dirichlet(\alpha )$.
(2) Choose a multinomial distribution $\varphi_{t}$ for the topic t from a Dirichlet distribution with parameter β, i.e., $\varphi_{t}∼Dirichlet(\beta )$.
(3) For a word $w_{k}$ in the document d, select a topic $z_{n}$ from a multinomial distribution $\theta_{d}$, i.e., $z_{n}∼Multi(\theta_{d})$, and select a word $w_{k}$ from a multinomial distribution $\varphi_{z_{n}}$, i.e., $w_{k}∼Multi(\varphi_{z_{n}})$. The probabilistic model is shown in Figure 3.

The modeling process of LDA can be described as a mixture of finding topics for each resource, namely each word in a document is obtained by selecting a certain topic with a specific probability and selecting a certain feature word from the topic with a certain probability, which can be formalized as Equation (3):

(3) $$P(w_{k}{|d}_{i})=\sum_{n=1}^{T}P(w_{k}|z_{n})P(z_{n}|d_{i})$$

where $P(w_{k}{|d}_{i})$ denotes the probability that the k-th feature word in a given document $d_{i}$ is selected. $z_{n}$ is a latent topic whose number is predetermined, and $P(w_{k}|z_{n})$ is the probability of the feature word $w_{k}$ occurring within topic $z_{n}$. $P(z_{n}|d_{i})$ is the probability of picking a feature word from topic $z_{n}$ in the document $d_{i}$.

##### Specialist Short-Term Knowledge Feature Topic Modeling

The strategy of making specialist recommendations by finding similar patients is one-sided, and there may be other specialists in the system that meet the needs of the target patient. Mapping specialist knowledge features to the latent topic space and finding specialists with similar probability distributions under the same topic can extend the recommended specialist candidate set at the semantic level. LDA topic model is used to cluster EMRs diagnosed by specialists, from which latent topics representing disease categories are identified. These topics indicate the disease features in which the specialist specializes and each specialist belongs to one or more latent topics. Then a topic-based description framework for specialist short-term knowledge features is generated. The specialist feature model based on short-term knowledge is developed on the latent topic space and its modeling process is divided into the following three steps.

**Step 1.** The EMRs diagnosed by specialists are used as the LDA training corpus, the latent topics $topic(t)=\{topic_{1},topic_{2},\ldots ,topic_{k}\}$ and the “document-topic” distribution of each specialist $d.topic\_profile=\{t_{1},t_{2},\ldots ,t_{k}\}$ are extracted by topic clustering, where k is the number of topics clustered by LDA.

**Step 2.** The LDA topic clustering generates a probability distribution of “topic-word” that can be used to characterize the short-term knowledge of specialists $d.feature\_profile=\{<f_{i},\omega_{i}>,i=1,2,\ldots ,n\}$, where $f_{i}$ is the feature word, $\omega_{i}$ is the weight of $f_{i}$, and n is the number of feature words.

**Step 3.** Based on the similarity of the “document-topic” probability distribution, we obtain specialists with similar knowledge features to those in the recommended specialist candidate set, and these similarities are regarded as the recommended index short_score for the short-term knowledge features of specialists, who also have the ability to treat the target patient.

In the LDA model, the text is measured by a topic probability vector from Dirichlet distribution, and the advantage of the topic model is weakened if the cosine similarity is used to calculate the text similarity. Kullback–Leibler (KL) divergence, a way to quantify the difference between two probability distributions [27], is used to calculate the similarity of document-topic distribution vector. However, KL divergence cannot be used as a distance measurement due to its asymmetry, that is, $D_{KL}(P\|Q)\neq D_{KL}(Q\|P)$. Thus, as deformation of KL divergence, Jensen–Shannon (JS) divergence with symmetry is proposed to compensate for deficiency of KL divergence, $D_{JS}\in [0,1]$. Smaller $D_{JS}$ values indicate more similar distributions. Hence, to facilitate the similarity calculation, the JS divergence value is converted to calculate the similarity of the “document-topic” probability distribution in this paper. The detailed procedures are shown in Appendix C.

### 3.4. Hybrid Recommendation Modeling

This section presents the construction process of the hybrid recommendation model. First, a professional recommendation method based on patients’ EMRs is constructed by integrating specialists’ activity to intelligently recommend relevant and active specialists for patients (called “professional recommendation”). Then, patients’ perceived utility feedback is introduced. The patient’s subjective Quality of Service (QoS) feedback assigns different preference weights to the professional recommendation and service quality to achieve an interpretable recommendation model, and dynamically adjusts the specialist recommendation index according to the patient’s objective QoS feedback results to further adjust and optimize the recommendation results.

#### 3.4.1. Professional Recommendation Integrating Specialists’ Activity

The frequency of telemedicine services performed by specialists can be considered as their explicit feedback on telemedicine, reflecting specialists’ activity. As the frequency of consultations increases, specialists show a higher level of activity in telemedicine information platforms. These specialists are more trusting and willing to serve telemedicine patients, which enables us to dynamically measure specialists’ activity in telemedicine based on the frequency. Therefore, besides the similarity index, specialists’ activity in the platform should also be considered, making the recommendation results biased toward specialists with higher activity and enthusiasm. Meanwhile, specialists’ activity will change over time. This paper adopts the attenuation function to model specialists’ activity, dynamically considering the frequency and time of consultations, which is expressed in Equation (4):

(4) $$AC_{j}=\sum_{t}\frac{N_{j}(t)e^{-t}}{N(t)}$$

where $N_{j}(t)$ represents the consultation frequency of specialist $S_{j}$ in period t, and N(t) is the total number of consultations in period t.

Furthermore, this paper converts the recent activity of specialists as in Equation (5) to reduce the leap in specialists’ activity, and $AC_{\max}$ denotes the activity of the most active specialist:

(5) $$LAC_{j}=\frac{AC_{j}}{AC_{\max}}$$

As mentioned above, the recommendation is made by integrating specialists’ activity on the basis of the specialist recommendation index, so that the distribution of recommendation results is biased towards the nearest and most frequent specialists [28]; the professional recommendation index is shown in Equation (6):

(6) prof_score=LAC×ini_score×short_score

#### 3.4.2. Hybrid Recommendation with Feedback Adjustment Mechanism

Hybrid recommendation incorporates the professional recommendation strategy and patient feedback evaluation into a single framework. User feedback is an essential part of the closed-loop control in the demand and service matching recommendation. This paper divides the patient feedback into subjective QoS feedback and objective QoS feedback.

##### Subjective QoS Feedback

Subjective QoS feedback refers to patients’ preference feedback on various recommendation indices before obtaining the recommendation result. The hybrid recommendation index is adjusted according to patients’ preferences to optimize the recommendation ranking, so that the recommendation results focus on high-weight content and form an interpretable recommendation strategy. After normalizing the recommendation indices, the above recommendation indices are linearly fused and expressed as follows:

(7) $$compre\_score=\omega_{p}prof\_score^{\prime }+\omega_{q}qos\_score$$

where $prof\_score^{\prime }=prof\_score/prof\_score_{\max}$, $\omega_{p}$ and $\omega_{q}$ represent the patient preference weights for the professional recommendation strategy and service quality, respectively, satisfying $0\leq \omega_{p},\omega_{q}\leq 1$ and $\omega_{p}+\omega_{q}=1$. In practice, we can reduce the computational complexity and time by asking patients simple questions about their recommended preferences, and then patients’ preferences can be converted into corresponding numbers, as shown in Table 2.

Patients’ preferences for different recommendation ways can be measured using the above conversion rules. For example, a patient selects “Extremely like” for professional recommendation, and “Like” for service quality, resulting in the corresponding values 4 and 3, then $\omega_{p}=4/7$, and $\omega_{q}=3/7$.

##### Objective QoS Feedback

Objective QoS feedback refers to patients’ post-evaluation after the completion of services. The patient evaluates the medical service based on their perceived quality in the service process, which demonstrates the patient’s satisfaction degree with the medical service and the specialist. For example, $qos_{1,j},qos_{2,j},\ldots ,qos_{m,j}$ are the comprehensive objective QoS evaluation value of the specialist $S_{j}$ by m patients, and the feedback evaluation of $qos_{i,j}$ is made by the patient $P_{i}$ after the completion of the service. Then the objective QoS value of specialist $S_{j}$ is further adjusted and updated by patients’ feedback as follows:

(8) $$qos_{j}=\frac{\sum_{u=1\neq i}^{m}qos_{u,j}+qos_{i,j}}{m+1}$$

It is converted into the patient feedback perceived utility index after normalization, as shown in Equation (9):

(9) $$qos\_score_{j}^{\prime }=\frac{qos_{j}}{qos_{\max}}$$

where $qos_{\max}$ denotes the highest evaluation value of all specialists. Finally, specialists’ QoS values are updated, and the ranking of the recommendation results is further adjusted and optimized by feedback.

## 4. Experimental Analysis and Evaluation of Results 

### 4.1. Sample Selection and Data Processing

All experiments were conducted with the data obtained from the National Telemedicine Center of China, which is operated by the First Affiliated Hospital of Zhengzhou University. There is no existing recommendation system on this platform. The different settings of medical institutions lead to differences in the division of departments, making the applicant doctors uncertain and ambiguous about the department they are applying for. In this paper, Internal Medicine Department (IMD) and Surgical Medicine Department (SMD) were selected as the dataset for experimental analysis without considering the specific branches of departments, because they are two major departments in the medical field with diverse and overlapping categories and a large amount of data.

**Figure 4:**
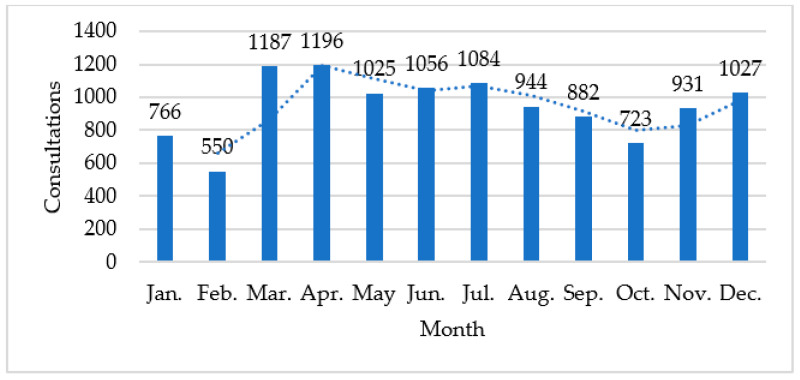
Distribution of consultations.

Before data integration, raw data were divided into these two departments. According to the department division published on the official website of the hospital, specific departments were divided into 12 major categories, such as IMD, SMD, Comprehensive Medicine Department, Gynecology Department, and Geriatrics Department. Then, the telemedicine records of specialists affiliated with IMD and SMD were extracted. To fully protect patient privacy, this paper compressed the data space as far as possible, and extracted the dataset containing four attributes: consultation time, diagnosis results, specialist name, and departments. A total of 11,371 annual data for 2020 were collected. The data statistics of the dataset are reported in Table 3, and the distribution of monthly consultations and its fitted curves are shown in Figure 4. Finally, specialists’ profiles were scraped from an online healthcare platform in China (https://www.haodf.com/, accessed on 8 January 2022) according to specialists’ names to comprehensively depict specialists’ knowledge backgrounds.

Since the directly acquired text information is crude, data preprocessing is required. First, synonymous disease names should be replaced with specific terms in the medical field, for example, “HBV” for “hepatitis B”, to ensure consistency of data. Meanwhile, the frequency statistics mentioned in Section 3.3 are adopted to fill the missing values to ensure the integrity of the data. Second, the Jieba package in Python is used for Chinese word segmentation, and we employ the Chinese medical thesaurus of Sogou Input [29] to construct a user dictionary to identify professional medical vocabulary during word separation processing. Finally, a stop word dictionary is created to eliminate stop words and filter out meaningless and useless words, numbers, and symbols to support text vectorization.

### 4.2. Experimental Design and Evaluation Metrics

#### 4.2.1. Experimental Design

Several experiments were designed to evaluate the performance of the method proposed in this paper. These experiments mainly focus on three aspects. (a) Experimental validation. The number of topics K significantly affects the clustering effect of the LDA topic model. The model perplexity under the different number of topics was counted to determine the optimal K to realize the optimal modeling performance of LDA. (b) Results analysis. The experimental results were discussed and analyzed to verify the feasibility of the proposed method. (c) Comparative analysis. The performance of the hybrid method proposed in this paper was compared and analyzed with the baseline method. Finally, the Accuracy, Recall, Relevance, and Activity of the recommendation results with different subjective QoS feedback from patients and different numbers of recommendation items were calculated.

#### 4.2.2. Evaluation Metrics

We employed Accuracy and Recall [30], widely used in top-N recommendation systems, as evaluation metrics for recommendation performance, and further tested the model performance through comparative analysis of Relevance and Activity of recommendation results. Accuracy (Pre@N) refers to the proportion of correctly recommended items to all recommended items. Recall (Rec@N) indicates the proportion of correctly recommended items to the items that should have been retrieved in the sample. These evaluation metrics are calculated as in Equations (10) and (11):

(10) $$Pre@N=\frac{TP}{TP+FP}$$

(11) $$Rec@N=\frac{TP}{TP+FN}$$

where TP is a true positive, FP is a false positive, FN is a false negative, namely incorrect items are recommended. The higher the Accuracy and Recall, the better the recommendation performance of the model.

Relevance refers to the similarity between the recommended specialist’s knowledge background and the target patient’s EMR; a higher similarity indicates that the specialist is more suitable to provide medical services for the target patient, and its calculation is shown in Equation (1). Activity refers to the enthusiasm of specialists in telemedicine activities as expressed in Equation (3).

### 4.3. Experiments and Result Analysis

#### 4.3.1. Topic Model Parameter Selection

**Figure 5:**
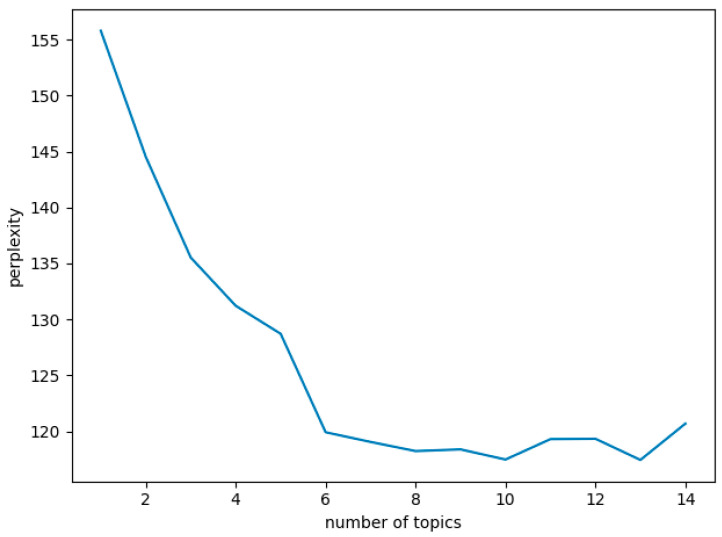
Perplexity.

To obtain a better model, the model parameters should be determined first. For the topic model, the number of topics is critical to the modeling quality and topic generation, but the number of topics is predetermined. If the number of topics is given by prior knowledge directly, the performance of the LDA model may not be optimal and greatly affect the recommendation effect, thus a scientific means should be adopted to select the number of topics. In this paper, perplexity is used to determine the number of topics, and the experimental results are shown in Figure 5. According to the elbow method, it can be seen that the LDA model has the lowest perplexity when K = 10; therefore, setting topics = 10 and iterations = 500, and presenting the high-frequency words (top-10) in the topic-word distribution of each topic.

#### 4.3.2. Experimental Results Analysis

We selected a specific patient (No. 1) from the dataset as the target to conduct the recommendation to validate the feasibility of the proposed method in this paper. First, after data integration and pre-processing, the patient’s EMRs were vectorized, and the similarity between the target patient’s EMRs and those of other patients was obtained by text mining, and the results are shown in Table 4.

Second, specialists with less than three consultations are defined as newly registered specialists, and the knowledge similarity between the newly registered specialists and the candidate specialists is obtained after the knowledge view similarity analysis to update the newly registered specialist recommendation index and the recommended specialist candidate set. As described in Section 3.3.1, $Sim_{Knowledge}(S162,S165)>0.7$, then the initial recommendation index of S162 was updated to $ini\_scor{e^{\prime }}_{162}=0.3137$, and the recommended specialist candidate set was updated to {S267, S183, S207, S184, S165, S275, S162, S94, S263, S120, S104}.

After that, the LDA topic model divided the historical diagnostic information of each specialist according to the document-topic distribution, and obtained specialists similar to the recommended candidate specialist according to the similarity of the distribution. The results are shown in Table 5.

Finally, the specialists’ activity and patient perceived utility were calculated and incorporated into the recommendation index. The hot-ranking recommendation is the result of fusion calculation based on multi-indicators such as patient voting, doctor response rate, word of mouth, and patient satisfaction according to certain rules, which can comprehensively express the service quality of doctors. Therefore, to facilitate the calculation, this paper selected the doctor’s comprehensive hot-ranking score on an online healthcare platform (https://www.haodf.com/, accessed on 8 January 2022) as the initial objective QoS value of each specialist. Then the recommended specialists were obtained by linear fusion of professional recommendation and patient feedback utility. The recommendation results are {S64, S3, S165, S67, S267, S183, S103, S207, S81, S184}, where the fusion coefficient is $\omega_{p}=0.6$, $\omega_{q}=0.4$.

We successfully recommended 10 specialists to the target patient, all of whom have extensive clinical experience in treating diabetes, hypertension, and respiratory acidosis, and obviously fit well with the patient’s treatment needs. The first recommended specialist is S267, whose labels include hypertension, diabetes and its complications, indicating that the specialist has extensive expertise in the field of endocrinology and metabolic diseases. The overall score is 4.5, reflecting the good feedback from her patients. The specialist has also demonstrated a high level of motivation in the telemedicine information platform. The above evidence validates the feasibility of the proposed approach to recommend relevant and active telemedicine specialists for patients who meet their needs. Moreover, it can be found that the final specialist recommendation results are significantly different from the initially recommended specialist candidate set. Although newly registered specialists were not recommended in this experimental strategy, a review revealed that newly registered specialists were recommended in another strategy, for example, $\omega_{p}=0.9$ and $\omega_{q}=0.1$, which illustrates that the proposed approach increases the recommendation chance of newly registered specialists to some extent.

#### 4.3.3. Comparative Experiments

To verify the scientificity and effectiveness of the proposed method, the dataset of 31 December 2020 was selected as the test data to evaluate the performance of the hybrid recommendation model by comparing experiments in different contexts. A recommendation is considered correct if a specialist’s profile includes the target patient’s disease label, that is, $\forall w_{k}\in d_{p_{i}}$, if $\exists w_{k}\in d.feature\_profile$, k=1,2,…,f, then return true, indicating that the specialist is competent to provide medical services to the patient $p_{i}$.

##### Validity Test of Weights for the Hybrid Recommendation

**Figure 6:**
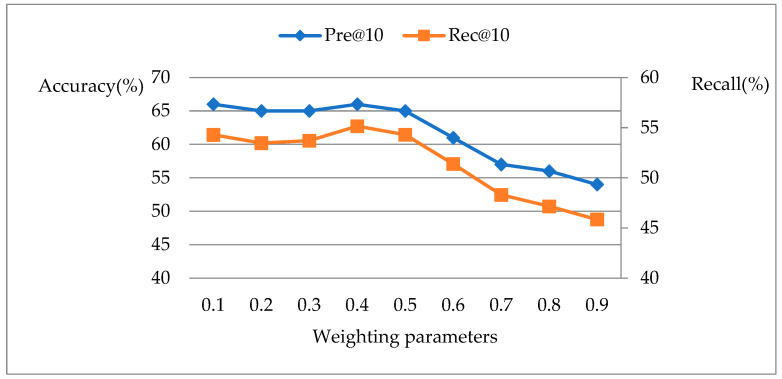
Performance of the hybrid recommendation model with different preferences.

The recommendation performance results of the hybrid recommendation model with the different weight $\omega_{q}$ are shown in Figure 6, where N = 10 denotes the number of results returned by the recommendation; the abscissa indicates the different values of $\omega_{q}$; the primary ordinate represents the Accuracy of the specialist recommendation, and the secondary ordinate represents the Recall of the specialist recommendation.

As can be seen from Figure 6, the Accuracy and Recall of the hybrid recommendation model maintain high level when $\omega_{q}\leq 0.4$. Later, as the weight $\omega_{q}$ increases, the Accuracy and Recall gradually decrease. The increase of $\omega_{q}$ means that patients attach significance to the service quality, which weakens the influence of the realistic background of specialists and patients on the recommendation results, and thus affects the overall performance of the recommendation model. Therefore, the patient perceived utility should not be overemphasized when making specialist recommendations.

##### Validity Test of Recommendation Items for the Hybrid Recommendation

**Figure 7:**
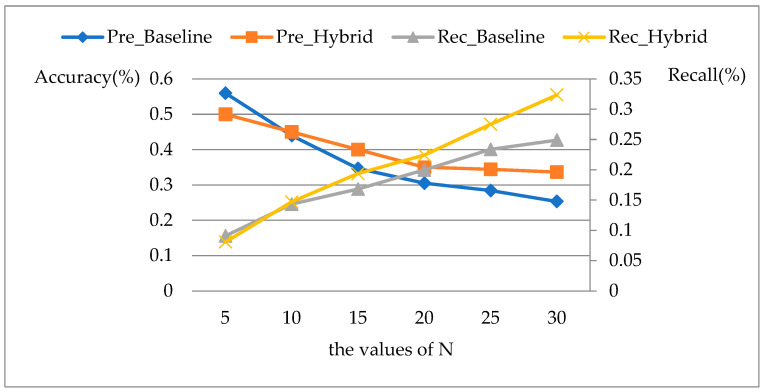
Comparison of Accuracy and Recall results.

The specialist recommendation requires recommending suitable specialists for target patients to address their health problems based on historical data of similar patients, which is a typical content-based recommendation method. Therefore, we adopt the content-based recommendation method as the baseline method to test the performance of the hybrid recommendation model. The experiment models the baseline method and the proposed hybrid recommendation method respectively, and evaluates the model performance by analyzing the Accuracy, Recall, Relevance, and Activity of these two models with different recommendation items. As stated above, the optimal experimental performance can be achieved when $\omega_{q}=0.4$. Therefore, we set $\omega_{p}=0.6$ and $\omega_{q}=0.4$ during the comparative analysis. The comparative experimental results of the model in terms of Accuracy and Recall are shown in Figure 7, where the abscissa indicates the number of specialist recommendation items.

According to the definition of Accuracy, in general, for the same algorithm, the larger the value of N, the lower the Accuracy of the recommendation model [20], that is, the highest accuracy is achieved when N = 5. When N > 5, pre_Hybrid>pre_Baseline, indicating that the hybrid method improves the accuracy of recommendation results. As for the Recall, it tends to increase as the value of N increases. Similarly, when N > 5, rec_Hybrid>rec_Baseline. In conclusion, the hybrid method proposed in this paper improves the accuracy and recall of specialist recommendation results.

**Figure 8:**
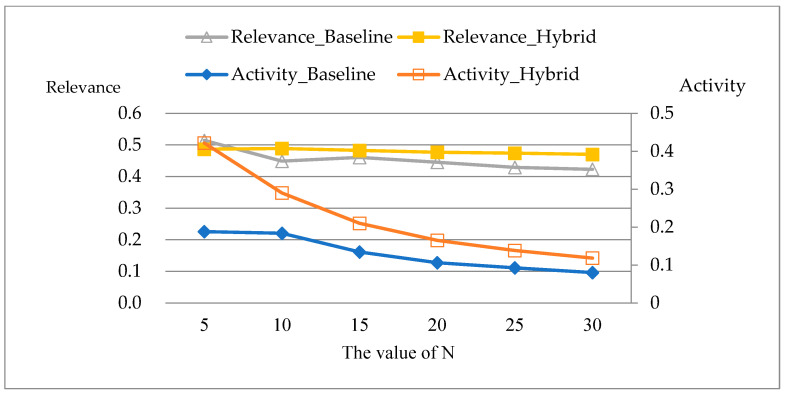
Comparison of Relevance and Activity.

Figure 8 illustrates the comparison of Relevance and Activity of recommendation results between the baseline and the hybrid model. The abscissa expresses the number of recommendation items, the primary ordinate expresses the Relevance of recommendation results, and the secondary ordinate expresses the Activity of recommendation results. From Figure 8, it can be seen that the recommendation results of hybrid recommendation outperform the baseline in terms of Activity. The Relevance gradually outperforms the baseline as the number of recommendation items increases, the reason for this is that the baseline completely relies on the similarity of patients’ EMRs for recommendations, and shows higher relevance when the number of recommendation items is small.

##### Rationality Evaluation of Recommendation Results

**Figure 9:**
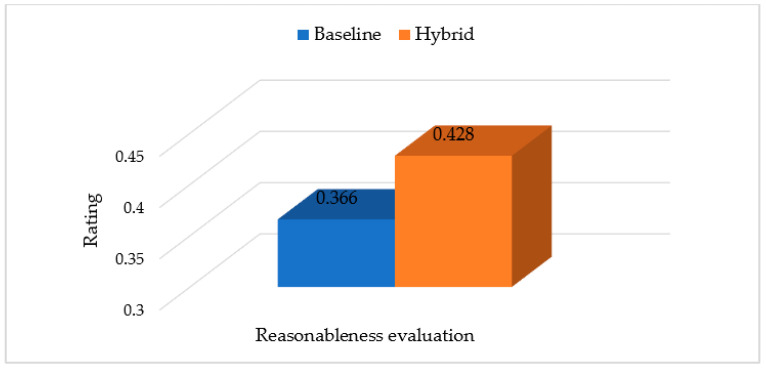
The rationality evaluation of the two recommendation methods.

To further test the performance of the hybrid recommendation strategy, the baseline model and the hybrid model were adopted to recommend specialists for a random patient, respectively, and a questionnaire was developed to investigate the rationality of the recommendation results. The questionnaire includes the target patient’s EMR and an assessment of the rationality of each recommended specialist, for example, “Please evaluate the matching level between the specialist and the target patient based on your work experience and the specialist’s activity. Is it reasonable to recommend the specialist $S_{1}$ to the target patient?”. The questionnaire was measured with a five-point Likert scale, rating from 1 (strongly unreasonable) to 5 (strongly reasonable). Furthermore, we set the priority for the ranking, $prio_{r}=(10-r)/\sum_{r=0}^{9}r$ which denotes the priority of the r-th specialist, then the overall rationality score of the r-th specialist is $score_{r}=prio_{r}\times \frac{1}{t}\sum_{d=1}^{t}score_{r}^{d}$, where $score_{r}^{d}$ denotes the rating of the d-th scheduler to the r-th specialist. The questionnaire was distributed to five staff members of the National Telemedicine Center of China who have long been engaged in telemedicine scheduling. The schedulers rated the rationality of all recommended specialists according to their own work experience, and the overall rating results are shown in Figure 9.

As shown in Figure 9, the hybrid recommendation method outperforms the baseline in terms of rationality evaluation, which is sufficient to prove that the specialists recommended by the hybrid recommendation method better meets patients’ disease and medical needs.

Overall, the hybrid recommendation can recommend relevant and highly motivated specialists for patients. In other words, the method proposed in this paper ensures the accuracy and relevance of the recommendations while considering the individual preferences of patients, as well as the high level of activity of the recommended specialists in telemedicine.

## 5. Conclusions and Future Works

### 5.1. Conclusions

This study uses the personalized recommendation technique to recommend appropriate telemedicine specialists for patients to reduce patient search costs and ensure the value of healthcare delivery. First, we construct specialist and patient feature models from patients’ EMRs and specialists’ long- and short-term knowledge backgrounds, respectively. Second, the initial recommendation index of a specialist is obtained by the similarity between patient features, and is supplemented by the similarity between specialists’ long-term knowledge profiles to update the newly registered specialist recommendation index. Then, the probabilistic topic model is adopted to establish the specialist short-term knowledge model in the topic space, and incorporate it into the recommendation index. Based on the above recommendation index, we propose a telemedicine specialist recommendation strategy that considers specialists’ activity and patients’ subjective and objective QoS feedback to achieve an adaptive telemedicine specialist recommendation. Finally, practical cases are compared and analyzed in terms of weight coefficients, number of recommendation items, and rationality evaluation to verify the effectiveness of the proposed method and its operability under the premise of data sparsity and privacy protection. This study provides the following contributions.

(1) The long- and short-term knowledge feature model of specialists is constructed. We use specialists’ profiles as the long-term knowledge to characterize their long-term accumulated experience, and the EMRs diagnosed by specialists in the telemedicine platform as the short-term knowledge to characterize the specialists’ recent concerns. The combination of the above two can describe specialists’ knowledge backgrounds comprehensively, and improve the accuracy and effectiveness of recommendations.
(2) The cold-start problem is alleviated using specialists’ long-term knowledge features. Based on the view similarity between specialists’ long-term knowledge features, we identify similar specialists to the initially recommended specialists and assign a newly recommended index to newly registered specialists accordingly. Then the initially recommended specialist set is updated and extended to increase the recommended chance of newly registered specialists and alleviate the cold-start problem to a certain extent.
(3) We propose a new metric, namely activity, to capture the motivation of specialists and incorporate it into the hybrid recommendation strategy. Specialists’ attitudes toward telemedicine are explained by the explicit behavioral feedback exhibited by specialists and its change over time, which reveals specialists’ activity in the telemedicine business. We propose a specialist recommendation method that considers activity, so that the distribution of recommendation results is biased toward the most frequent and active specialists, thereby improving the recommendation capability.
(4) The feedback adjustment mechanism is introduced into the recommendation strategy to realize the self-adaptive recommendation. The subjective QoS feedback adjusts patients’ preference weights for the professional recommendation and service quality to optimize recommendation ranking, so that the recommendation results focus on high-weight content, leading to an interpretable recommendation strategy. For example, when patients pay more attention to professionalism, the model focuses more on the matching of professional backgrounds, and the interpretation of its recommendation results is expressed as the matching of objective disease characteristics; when patients pay more attention to service quality, the model focuses more on the examination of the comprehensive service quality of specialists. Furthermore, the specialists’ QoS value can be adjusted through the objective QoS feedback of patients after the medical service is completed. Therefore, real-time closed-loop adjustment of specialist recommendations is carried out through subjective and objective QoS feedback mechanisms, making the recommendations time-sensitive while considering patient satisfaction.

This study also reveals some implications for managing healthcare platforms as well as other online platforms. The effectiveness of the proposed method has been proven, so that managers of platforms can apply the proposed method to alleviate the information overload confronted by patients and recommend the appropriate specialists for patients based on their preferences and needs. Information overload problems also exist in other online platforms. For example, consumers on e-commerce platforms are confused by the huge number of products. Drawing on the idea of our proposed method, e-commerce platforms can recommend suitable products to consumers. The method is also applicable to online health Q&A platforms and reviewer recommendation systems. By considering the interests of doctors or experts and their changes over time, the questions and review manuscripts that match the interests of doctors or experts are recommended to them, improving the rationality of recommendations and ensuring efficiency and effectiveness.

### 5.2. Limitations and Future Directions

However, there are some limitations to this study. Future work can extend this research from several aspects: (a) This study focuses on the matching between specialist service capacity and patient service demand without regard to the waiting cost. Future research needs to be further investigated together with specialist scheduling to develop a more accurate specialist recommendation model; (b) As the volume of data increases, a suitable method of compressing the computational space is necessary to improve computational efficiency; (c) Future research should review the correlation and interdependence of attributes between specialist service capacity and service quality.

## Figures and Tables

**Table 1:** Specialist Knowledge Attribute Matrix.

Specialists	Knowledge Attributes			
	$A_{1}$	$A_{2}$	…	$A_{g}$
$S_{1}$	$a_{11}$	$a_{12}$	…	$a_{1g}$
$S_{2}$	$a_{21}$	$a_{22}$	…	$a_{2g}$
…	…	…	…	…
$S_{n}$	$a_{n1}$	$a_{n2}$	…	$a_{ng}$

**Table 2:** Conversion rules between textual and numerical preferences.

**Textual Preference**	Extremely Like	Like	Fair	Dislike	Extremely Dislike
**Numerical preference**	4	3	2	1	0

**Table 3:** Data Statistics.

Dataset	#Specialists	#Patients	#Consultations
IMD	163	7598	8233
SMD	120	3010	3138

**Table 4:** Similarity of the target patient.

No.	#Patient	Similarity	#Specialist
1	9629	0.4661	S267
2	8730	0.4572	S183
3	10,444	0.4509	S207
4	771	0.4418	S184
5	987	0.3578	S165
6	1127	0.3216	S275
7	2333	0.3025	S94
8	6056	0.3025	S263
9	6771	0.3012	S120
10	6773	0.3012	S104

**Table 5:** Similarity of topic distribution.

Specialist	Similarity	Similar Specialist	…	Specialist	Similarity	Similar Specialist
S267	0.9837	S3	…	S104	0.9018	S268
	0.9796	S270			0.8976	S177
	0.9773	S67			0.8629	S25
	0.9640	S152			0.8579	S221
	0.9622	S63			0.8514	S225
	0.9436	S207			0.8109	S241
	0.9400	S8			0.8040	S175
	0.9246	S9			0.8007	S133
	0.9005	S200			0.7888	S87
	…	…			…	…

## Data Availability

Data sharing is not applicable to this article.

## Appendix A. Patient Feature Modeling

The TF-IDF value of the keyword $w_{k}$ in $d_{i}$ can be expressed as:

(A1) $$TF-IDF(w_{k},d_{i})=TF(w_{k},d_{i})\times IDFf(w_{k})$$

(A2) $$TF(w_{k},d_{i})=\frac{n_{k,i}}{\sum_{k=1}^{f}w_{k,i}}$$

(A3) $$IDF(w_{k})=\log\frac{m}{\left|\left\{d:w_{k}\in d\right\}\right|}$$

where $d_{i}$ denotes the EMR of $p_{i}$, $n_{k,i}$ denotes the frequency of feature word $w_{k}$ in the EMR $d_{i}$, $\left|\left\{d:w_{k}\in d\right\}\right|$ denotes the number of EMRs containing the feature word $w_{k}$. However, if an EMR does not contain the feature word $w_{k}$, the divisor will be 0, making the equation meaningless. Therefore, $\left|\left\{d:w_{k}\in d\right\}\right|$ is usually expressed as $1+\left|\left\{d:w_{k}\in d\right\}\right|$.

## Appendix B. Knowledge View-Based Long-Term Knowledge Feature Modeling

This paper used Jaccard similarity coefficient to calculate the attribute similarity between knowledge with the following Equation (A4):

(A4) $$Jaccard(j,h)=\frac{\left|A(j)\cap A(h)\right|}{\left|A(j)\cup A(h)\right|}$$

where A(j) and A(h) denote the set of knowledge attributes of $S_{j}$ and $S_{h}$, respectively. |A(j)∩A(h)| denotes the number of knowledges owned by both $S_{j}$ and $S_{h}$, and |A(j)∪A(h)| denotes the number of knowledges shared by $S_{j}$ and $S_{h}$.

The weighted Jaccard knowledge view similarity is expressed as Equation (A5):

(A5) $$Sim_{Knowledge}(j,h)=\frac{\sum_{a_{\alpha }\in A(j)\cap A(h)}\omega (a_{\alpha })}{\sum_{a_{\beta }\in A(j)\cup A(h)}\omega (a_{\beta })}$$

where ω(a) is the weight of the knowledge attribute.

To make full use of the attribute information, the attribute weights are determined by information entropy (as shown in Equations (A6) and (A7)), and the weights are learned from the data, avoiding the problem of excessive subjectivity.

(A6) $$\omega (a)=-p(a)\log_{2}p(a)-(1-p(a))\log_{2}(1-p(a))$$

(A7) $$p(a)=\frac{n(a)}{\left|S\right|}$$

where p(a) is the probability of occurrence of attribute a, and n(a) is the frequency of occurrence of attribute a.

## Appendix C. LDA-Based Short-Term Knowledge Feature Modeling

The KL divergence of two distributions (P and Q) can be expressed as:

(A8) $$D_{KL}(P\|Q)=\sum_{x\in X}P(x)\log\frac{P(x)}{Q(x)}$$

The JS divergence of two distributions (P and Q) can be formulated as:

(A9) $$D_{JS}(P\|Q)=\frac{1}{2}D_{KL}(P\|\frac{P+Q}{2})+\frac{1}{2}D_{KL}(Q\|\frac{P+Q}{2})$$

where $D_{JS}\in [0,1]$, and smaller JS values indicate more similar distributions, and vice versa. When the two distributions are identical, $D_{JS}=0$. Therefore, to facilitate the similarity calculation, the JS divergence value is converted in this paper, as shown in Equation (A10), where ε is the moderator parameter and the value range of similarity is [0, 1].

(A10) $$short\_score=Sim_{Doctor}(P,Q)=\frac{1}{1+D_{JS}(P\|Q)\times \varepsilon }$$
